# Nano-Biomimetic Drug Delivery Vehicles: Potential Approaches for COVID-19 Treatment

**DOI:** 10.3390/molecules25245952

**Published:** 2020-12-16

**Authors:** Bwalya A. Witika, Pedzisai A. Makoni, Larry L. Mweetwa, Pascal V. Ntemi, Melissa T. R. Chikukwa, Scott K. Matafwali, Chiluba Mwila, Steward Mudenda, Jonathan Katandula, Roderick B. Walker

**Affiliations:** 1 Department of Pharmacy, DDT College of Medicine, P.O. Box 70587, Gaborone 00000, Botswana; bwitika@ddtcollegeofmedicine.com (B.A.W.); lmweetwa@ddtcollegeofmedicine.com (L.L.M.); 2 Division of Pharmaceutics, Faculty of Pharmacy, Rhodes University, Makhanda 6140, South Africa; p.makoni@ru.ac.za (P.A.M.); pvn8990@gmail.com (P.V.N.); m.chikukwa@ru.ac.za (M.T.R.C.); 3 Department of Basic Sciences, School of Medicine, Copperbelt University, Ndola 10101, Zambia; scott.matafwali@cbu.ac.zm; 4 Department of Pharmacy, School of Health Sciences, University of Zambia, Lusaka 10101, Zambia; chiluba.mwila@unza.zm (C.M.); steward.mudenda@unza.zm (S.M.); 5 Department of Biosciences and Chemistry, Faculty of Health and Wellbeing, Sheffield Hallam University, Sheffield S1 1WB, UK; Katandulajonathan@yahoo.com

**Keywords:** biomimetic drug delivery, SARS-CoV-2, COVID-19, nanotechnology, cytokine storm syndrome, nanomedicine

## Abstract

The current COVID-19 pandemic has tested the resolve of the global community with more than 35 million infections worldwide and numbers increasing with no cure or vaccine available to date. Nanomedicines have an advantage of providing enhanced permeability and retention and have been extensively studied as targeted drug delivery strategies for the treatment of different disease. The role of monocytes, erythrocytes, thrombocytes, and macrophages in diseases, including infectious and inflammatory diseases, cancer, and atherosclerosis, are better understood and have resulted in improved strategies for targeting and in some instances mimicking these cell types to improve therapeutic outcomes. Consequently, these primary cell types can be exploited for the purposes of serving as a “Trojan horse” for targeted delivery to identified organs and sites of inflammation. State of the art and potential utilization of nanocarriers such as nanospheres/nanocapsules, nanocrystals, liposomes, solid lipid nanoparticles/nano-structured lipid carriers, dendrimers, and nanosponges for biomimicry and/or targeted delivery of bioactives to cells are reported herein and their potential use in the treatment of COVID-19 infections discussed. Physicochemical properties, viz., hydrophilicity, particle shape, surface charge, composition, concentration, the use of different target-specific ligands on the surface of carriers, and the impact on carrier efficacy and specificity are also discussed.

## 1. Introduction

Towards the end of 2019, a sudden acute atypical respiratory disease was identified in the Wuhan province of China, with most initial cases identified to have been exposed at the Huanan seafood market at which the sale of dead seafood and live animals occurred [1]. The Chinese government notified the World Health Organization (WHO) and closed the Huanan seafood market in January 2020. A drastic increase in the number of cases has been observed subsequently, including persons who had not been exposed to the seafood market directly, which confirmed human to human transmission of the causative organism [2]. The disease initially spread to Thailand, South Korea, and Japan as a consequence of massive Chinese migration due to celebration of the Chinese New Year. An epidemic caused by a novel coronavirus, with the first fatality reported on 11 January 2020, had commenced [1]. The pathogen was ultimately identified as a novel enveloped RNA β coronavirus that was subsequently named severe acute respiratory syndrome coronavirus 2 (SARS-CoV-2) [2], and the SARS-CoV-2 tag was assigned due to the virus exhibiting approximately 80% homology to the SARS-CoV, which caused acute respiratory distress syndrome (ARDS) with associated high mortality during the early 2000 s [3].

In February 2020, WHO referred to the disease caused by this virus Coronavirus disease 19 or COVID-19, and a pandemic was declared in March 2020, the impact of which has been widespread, with >200 countries and territories being affected [4,5]. As of 5 November 2020, over 47 million cases have been recorded worldwide resulting in more than 1.2 million deaths. Of the over 36 million cases that have had an outcome, 97% have been reported as recoveries [5].

The primary site of infection of the SARS-CoV-2 virus has been reported as the respiratory system possibly due to the vast surface area of the lungs that makes them highly susceptible to the virus, if inhaled [6], and subsequent pathology involves broad systemic infection. The symptoms associated with COVID-19 include lower respiratory tract infection and related symptoms, viz., dry cough, dyspnea, ARDS, and pulmonary fibrosis in addition to more general symptoms such as fever, headache, dizziness, generalized weakness, vomiting, and diarrhea [7]. In some instances, patients may experience fulminant and fatal hyper-cytokinemia associated with multi-organ failure (MOF) as a result of cytokine storm syndrome [8]. In addition, patients with COVID-19 may also experience cardiac, hepatic, renal, central nervous system, or thrombotic disease [9].

Current medical management of COVID-19 infection is largely supportive with no specific therapy available. Several drugs, including antimalarials such as chloroquine and hydroxychloroquine [10,11,12], the anti-retroviral combination lopinavir/ritonavir [13], an investigational nucleotide analog with broad-spectrum antiviral activity initially intended to treat hepatitis C and Ebola, viz., remdesivir [14], and the macrolide antibiotic azithromycin [11,12], have been tested in clinical trials as potential treatment for the virus. However, none of these approaches are a definitive cure or are suitable for prophylaxis.

Nanomedicines are treatment platforms made typically of particles designed in the nanoscale size range to deliver active pharmaceutical ingredients (APIs) with the intention of enhancing efficacy, safety, accuracy of diagnosis and/or adherence with targeted treatment of diseases [15]. The benefits of using nanomedicines may be realized using the unique properties of engineered nanomaterials, viz., their physicochemical properties, including size, shape, chemical composition, physiochemical stability, crystal structure, surface area, surface energy, and surface roughness and/or use of a variety of target-specific ligands on the surface(s) of these carriers [16]. For the purposes of this review, nanoparticles are defined as any particle that exhibits nanoscale dimensions, i.e., 1–1000 nm. The biomaterials used in the fabrication of nanomedicines must exhibit biocompatibility to minimize potential harmful effects to patients in order to provide efficacy without adverse events. In addition, appropriate biomaterials must be selected to ensure the adequate delivery of the payload following administration, necessitating confirmation through quality control process of target critical quality attributes (CQA) following manufacture [17]. Nanomaterials based on bioinspired synthesis have been developed with the primary aim of simulating the unique properties of naturally occurring structures of organisms and associated biosynthetic pathways [18]. The biomimetic delivery vehicles for which the morphology, surface properties, and/or size resemble/mimic natural structures of organisms and cell lines, such as macrophages [19], erythrocytes [20], thrombocytes [21], exosomes [22], or pathogens [23,24], exhibit special functions for the enhancement of delivery to target tissue or cell populations.

**Figure 1:**
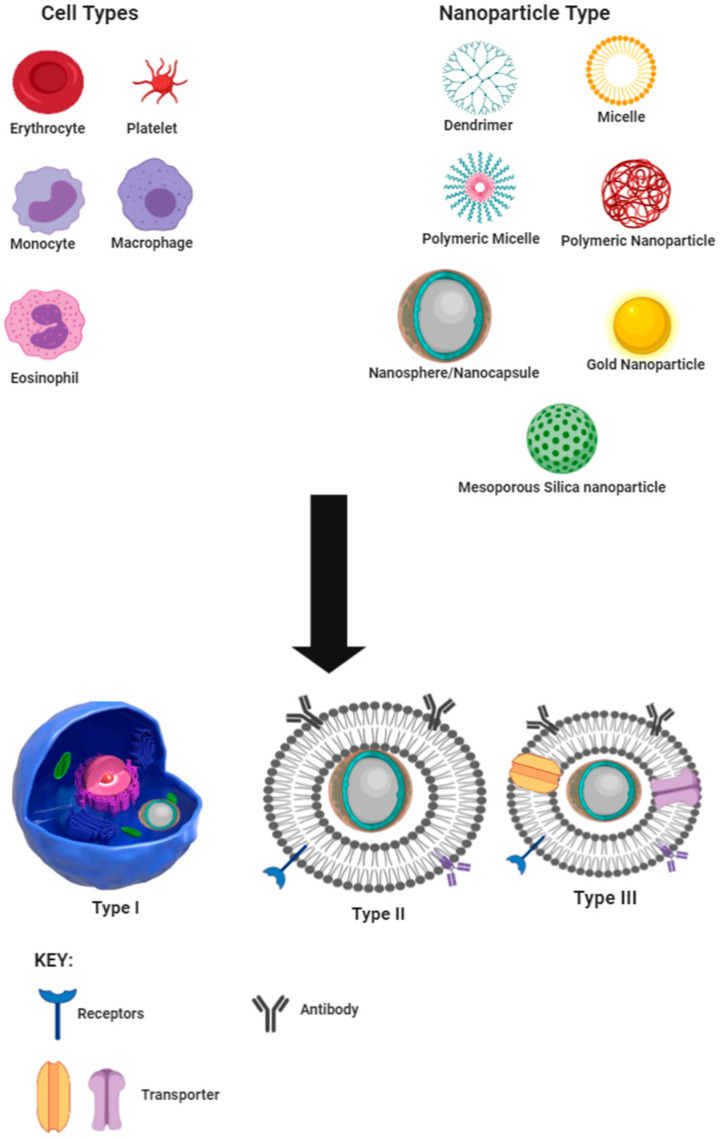
Schematic representation of types and sources of cell-derived biomimetic nano-drug delivery systems.

In this review, we categorize biomimicry into three types, viz., I, II, and III. These definitions, which are closely adapted to previously described classifications [25], are schematically depicted in Figure 1 using nanospheres as an example and are used in this review as defined vide infra.

Type I, also known as the cell, involves encapsulation of nanoparticles into a cell or pathogen for shuttling the nanoparticle to the site of action and has been suggested as a treatment option for accessing the human immunodeficiency virus (HIV) with macrophages to treat central nervous system infection [26,27,28]. Type II or sub-membrane transfer systems make use of parts of cells, such as specific receptors, in order to detect receptor-specific substrates or perform the role of the cell component attached. This approach has been used for target/ligand-specific techniques for treating cancer using liposomes as the carrier technology [29,30]. Type III or total cell membrane transfer systems make use of whole membrane removal from cells and encapsulation of the nanocarrier with the cell membrane so as to mimic the cell from which the membrane is harvested.

## 2. COVID-19 Pathogenesis

### 2.1. Initial Infection

**Figure 2:**
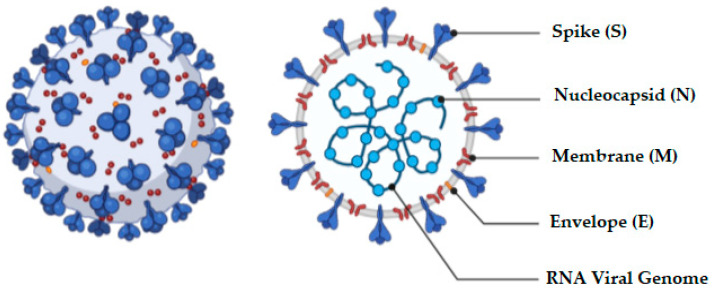
Schematic representation of the structure of the coronavirus.

The coronavirus is a an enclosed, positive-sense, single-stranded RNA virus of approximately 30 kb capable of infecting a wide variety of host species [31]. The virus has four structural components, viz., spike, nucleocapsid, envelope, and membrane proteins [32], and the appearance of the virus and associated proteins are depicted in Figure 2.

Four genera of the coronavirus, viz., α, β, γ, and δ, exist and are differentiated on the basis of their genomic structure. Of these, the α and β infect mammals [32] and humans, and NL63 and 229E cause croup and cold, which are classic symptoms of infections by the α genus. The inhaled virus binds to epithelial cells in the nasal cavity and disseminates and migrates down the respiratory tract to the lungs [33]. Distribution and expression of receptors lead to regulation of tropism, thereby initiating pathogenesis of the disease [34]. The life-cycle of the virus is comprised of five stages and include attachment, penetration, bio-synthesis, maturation, and finally release [35]. Immediately following virus attachment to host receptors, penetration occurs through a process of membrane fusion and/or endocytosis. The viral RNA is subsequently released into host cells, where it replicates in the host cell nucleus resulting in biosynthesis of viral proteins. New particles of virus then mature and are released into the host. Coronavirus entry into host cells is an important determinant of viral infectivity and pathogenesis [36,37] and is also a major target for host immune surveillance and intervention strategies [36,38,39]. To enter host cells, coronaviruses first bind to surface receptors on the cell and subsequently enter an endosome, eventually resulting in fusion of the virus and lysosome membranes [36,37].

The composition of the spike (S) protein (Figure 2) includes a transmembrane tri-metric glycoprotein that protrudes extensively on the surface of the virus. The protrusion or spike is the primary determinant of the diversity and host tropism of the coronavirus and is further bifurcated into the functional sub-units S1 and S2 [32]. Specifically, sub-unit S1 is responsible for attaching the virus to the receptor of the host cell and sub-unit S2 for the fusion process with the cell membrane by the virus. Angiotensin-converting enzyme 2 (ACE-2) has been investigated as one of the functional receptors for SARS-CoV [40]. The entry of SARS-CoV-2 into the host cell is dependent on the presence of a 180-kDa spike protein that is mediated by two critical events: ACE-2 binding to the amino-terminal region of the spike protein, and viral fusion with cellular membranes through the carboxyl-terminal region of the spike [34]. Infection of pulmonary cells requires proteolytic activation of the spike protein by cleavage of polyo-basic furin [41]. The furin protease leads to expansion of SARS-CoV-2 tropism, which is assumed to have resulted in the transferal of the virus from bats to humans through an intermediary host [41]. In addition to the vast surface area of the lungs that make this organ a likely target for SARS-CoV-2, it has been shown that 83% of ACE-2-expressing cells in the human lungs are alveolar epithelial type II cells, suggesting that these cells may act as a portal for viral invasion [6,42]. Furthermore, gene ontology enrichment analysis has revealed that ACE-2 expressing alveolar epithelial type II cells exhibit high levels of regulatory genes for viral processes, life cycle, assembly, and genome replication suggesting that ACE2-expressing alveolar epithelial type II cells facilitate and aid replication of SARS-CoV-2 in the lungs [6,42].

### 2.2. Cellular Mechanism (Cascade) of COVID-19 Infection

**Figure 3:**
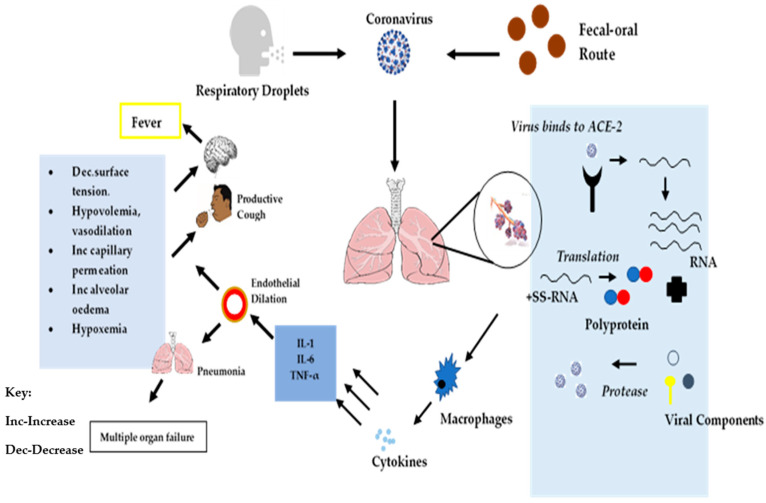
Schematic representation of SARS-CoV-2 cellular infection cascade and symptom onset.

ACE-2 is a trans-membrane protein that has been characterized for its homeostatic role in counterbalancing the impact of ACE on the cardiovascular system (CVD) [43]. Angiotensin I is converted to angiotensin II, a highly active octa-peptide that causes contraction of blood vessels to increase pressure and blood flow in addition to exhibiting pro-inflammation activities. ACE-2 activity of carboxypeptidase leads to the conversion of angiotensin II into hepta-peptide angiotensin, which is known as the functional antagonist of the angiotensin II enzyme [34]. The high expression of ACE in the endothelial cells of the vasculature in the lungs results in a high probability of the presence of angiotensin II within the lung cells, which contributes to interference with pulmonary vasculature regulation [44]. Type-II alveolar pneumocytes directly mediate the innate activity of the pro-inflammatory response of the SARS-CoV-2 in the lower respiratory tract due to the presence of ACE-2 in these cells. Type-II pneumocytes function as cells capable of producing interleukin (IL)-6, tumor necrosis factor (TNF)-α, granulocyte macrophage colony-stimulating factor (GM-CSF), monocyte chemoattractant protein (MCP)-1, and IL-1β in the pulmonary system [45]. As illustrated in Figure 3, infected lung cells cause an increment in the levels of pro-inflammatory cytokines leading to endothelial dilation of alveolar cells, which is responsible for a decrease in the alveolar surface tension through accumulation of surfactant in pulmonary cells, hypovolemia, increased capillary permeability, alveolar edema, and hypoxemia [45]. Infected pulmonary tissues are also an indirect mechanism that induces multi-system organ dysfunction, which is characterized by acute lung failure, acute kidney injury, acute liver failure, cardiovascular diseases, as well as a wide spectrum of the hematological abnormalities, including neurological disorders [44]. In addition, the presence of IL-6, IL-1β, and TNF-α has an effect on the hypothalamus region of the brain, which controls body temperature [34] and which may induce fever, which is a potential symptom of coronavirus infection. The possible infection cascade and classification based on pathological manifestation is depicted in Figure 3.

### 2.3. Mild-to-Severe Pathological Manifestations

The clinical manifestations and associated stages of the COVID-19 disease are summarized in Table 1. In mild-to-severe cases of COVID-19 infection, patients may present with fever, dry cough, sore throat, headache, fatigue, chest pain, dyspnea, muscle pain, gastrointestinal distress, nausea, and/or vomiting [32,46]. The virus may be detected in the lower respiratory tract in patients who present with these symptoms, and pronounced radiological changes in pulmonary tissues may be evident. Histological examination reveals that patients suffering with a mild case of the disease present with diffuse alveolar damage, exudation of fibrin, proliferation, and desquamation of type II alveolar epithelial (type II AE) cells, formation of hyaline membranes, and the presence of macrophages and monocytes [47]. Lung consolidation, pulmonary opacity, damage to the alveolar septa, presence of monocytes, and lymphocytes have also been reported following chest computed tomography (CT) scanning [48]. In patients presenting with moderate disease, pneumonia accompanied by frequent fever and cough is evident, while in severe cases, patients present with pneumonia and hypoxemia [32]. When patients are in critical condition, they present with ARDS, shock, myocardial injury, encephalopathy, heart failure, coagulation dysfunction, and acute kidney injury [32].

## 3. Pharmacological and Cellular Targets for Biomimetic Drug Delivery

Currently, no cure for SARS-CoV-2 exists; however, several possibilities of a cure can be postulated and developed for the prevention of the existing threat of SARS-CoV-2. Many of the current therapeutic strategies are based on repurposing of existing drugs, and only a few are in development, specifically for mitigating the current pandemic [49]. Therapeutic options include the use of peptides, small molecule drugs, monoclonal antibodies, interferon, and vaccine approaches. From a pharmacological perspective several targets for interruption of the life cycle of the coronavirus may be explored, including pre- and post-entry stages of infection. The targets for interrupting the life cycle can be used to develop potential therapeutics that inhibit viral pathogenesis of SARS-CoV-2 and the use of engineered nanocarriers to deliver these therapeutic candidates safely and effectively explored.

The steps in the lifecycle of the virus, which are potential targets for drug therapy, require evaluation of biomimetic drugs that are able to target cellular activities such as blocking of SARS-CoV-2 entry, endocytosis and fusion with the cell membrane, inhibition of viral enzymes, suppression of inflammation, and inhibition of viral components, including viral envelope, membrane, nucleocapsid, and/or accessory proteins [49,50,51].

### 3.1. Blocking of Fusion and Entry of SARS-CoV-2 into Cells

#### 3.1.1. ACE-2/S-Protein-Receptor Domain Binding Interactions

The SARS-CoV-2 virus makes use of spike proteins present on the surface of the viral envelope to enter host cells [52,53]. This interaction between the spike proteins and ACE-2 receptors are a potential pharmacological target for treatment of infections. Engaging the ACE-2 receptor with recombinant human derived ACE-2 is an approach that can be explored, as the delivery of excess soluble ACE-2 may neutralize the virus through competitive binding to the SARS-CoV-2 envelope [54,55]. In addition, viral entry into cells may be blocked by proteins, peptides, or small molecule compounds that bind to the S protein of the virus, thereby preventing interaction of the virus with the host cell membrane [51,56,57].

Since SARS-CoV-2 enters the host cell by binding to ACE-2 at the S protein receptor-binding domain (RBD) [58], inhibition of SARS-CoV-2 RBD/ACE2 protein–protein interaction (PPI) is potentially a very important therapeutic target. The first-in-class peptide binder, 23-mer peptide binder (SBP1), was found to potentially restrict the entry process of SARS-CoV-2 into human cells through binding to the SARS-CoV-2-RBD [59], suggesting this is a potential approach to treatment.

#### 3.1.2. Fusion

The entry of the SARS-CoV-2 organism to host cells is facilitated primarily by two host proteases, viz., serine protease TMPRSS2 on the surface of the cell surface, and/or cysteine proteases cathepsin B and L (CatB/L) in endosomes [60,61]. The development of protease inhibitors could be useful to target these proteases as treatment options for COVID-19. Camostat mesylate, aTMPRSS2 serine protease inhibitor and a cathepsin L inhibitor, are novel agents which may be used to inhibit COVID-19 entry into host cells. The cathepsin L inhibitor, in particular, has exhibited good results in reducing infections in human lung cell lines when administered concomitantly with camostat mesylate [50,62,63]. Nafamostat mesylate has also been shown to prevent TMPRSS2-triggered SARS-CoV-2 membrane fusion and has been licensed for use in Japan [61].

Unlike in SARS-CoV, the S protein of SARS-CoV-2 has a furin cleavage site at the S1/S2 boundary similar to that observed for the MERS-CoV organism [58,64] that facilitates virus entry and subsequent infection, potentially increasing viral transmission [65]. Targeting this unique furin-like cleavage site of the spike glycoprotein is a potential target that can be explored, and furin inhibitors such as decanoyl-RVKR-chloromethylketone (CMK) and naphthofluoresce that could halt SARS-CoV-2 pathogenesis in vitro and in vivo are currently being evaluated [66,67].

HR1 and HR2 present in SARS-CoV-2 facilitate cell membrane fusion [61], and peptides derived from HR2 bind to HR1, facilitating fusion of SARS-CoV-2 with host cells. Inhibition of SARS-CoV-2 by developing potent peptide-based inhibitors that specifically target the HR1–HR2 interaction at the S2 protein of the coronavirus are in development. HR1 and HR2 derived peptides such as the pan-coronavirus fusion inhibitor, EK1, designated for SARS-CoV-2, showed potent fusion inhibitory effects indicating that SARS-CoV-2–HR2P may be a promising therapeutic compound for treating SARS-CoV-2 infections [68].

### 3.2. Blocking Endocytosis

Targeting endocytosis is another potential strategy for developing potential candidates to treat SARS-CoV-2 infections, since the virus undergoes endocytosis in a pH- and receptor-dependent process following fusion with the host cell [61]. Several possible drug candidates including janus kinase (JAK) inhibitors such as baricitinib and ouabain, a clathrin medicated inhibitor, are undergoing clinical trials in SARS-CoV-2 positive patients [69]. Chloroquine and hydroxychloroquine have been evaluated for their ability to inhibit viral progression of SARS-CoV-2 [70,71]. The exact molecular mechanism of action of hydroxychloroquine for the treatment of infection remains elusive, but it is believed to be a consequence of endosome-mediated viral entry or late stage viral replication impairment [72]. However, the results of preliminary large-scale randomized controlled trials with chloroquine and hydroxychloroquine have yet to show survival benefits in COVID-19 treatment, with experts discouraging the use of these molecules for the treatment and/or post-exposure prophylaxis of COVID-19 [73,74].

### 3.3. Viral Enzyme Inhibition

Papain-like cysteine protease (PLpro) and 3C-like serine protease (3CLpro or Mpro) viral enzymes are implicated in the delivery of non-structural proteins, including RNA-dependent RNA polymerase (RdRp) and helicase, which are involved in the process of transcription and replication of the virus [75,76]. Potential therapeutic compounds which inhibit 3CLpro and PLpro may be explored for the treatment of COVID-19 infections.

Multiple drugs that have been developed for targeting protease, polymerase, and helicase in other viral pathogens are now being evaluated in clinical trials for treating SARS-CoV-2 and include remdesivir [14,77], favipiravir [78,79,80], and lopinavir/ritonavir [13,81]. Remdesivir is an experimental drug originally developed as an RNA dependent RNA polymerase (RdRP) inhibitor for treating the Ebola virus (EBOV) and has exhibited positive efficacy against COVID-19 in a phase 3 trial, and the USFDA has approved emergency use of remdesivir in the USA as have many other countries [82].

### 3.4. Suppression of Excessive Inflammatory Response

In some patients infected with the SARS-CoV-2 organism, a hyper-inflammatory response, possibly due to deregulated cytokine production, has been observed and is referred to as an inflammatory cytokine storm [82]. COVID-19 patients treated in intensive care units (ICU) have presented with extremely high levels of cytokine in plasma when compared to patients treated external to the ICU, suggesting that dysregulation of the cytokine response occurs in the severe form of COVID-19 disease [83,84]. Furthermore, SARS-CoV-2 infected patients admitted to the ICU present with increased GM-CSF and IL6+CD4+T cells when compared to patients not yet admitted to the ICU [85,86]. Therefore, inhibition of excessive inflammatory response may reduce the severity and morbidity of COVID-19 disease. Corticosteroids have been used to suppress systemic inflammation [49,87], and dexamethasone has proved beneficial when used to treat critically ill COVID-19 patients, and reduced mortality has been observed [49,88]. Therapeutic agents such as tocilizumab, which can bind specifically to soluble IL-6 and membrane-bound IL-6 receptors, thereby inhibiting signal transduction, may be the first IL-6-blocking antibody useful for treating COVID-19 infections [89].

### 3.5. Convalescent Plasma Treatment

Convalescent plasma (CP) therapy has been proposed as a potential treatment strategy for COVID-19 infections [90,91]. The plasma from a donor who has recovered from an infection is transfused in an attempt to develop passive humoral immunity against SARS-CoV-2-infections in patients. A study conducted by Salazar et al. [92] showed significant reduction in mortality (*p* = 0.047) was observed when CP from a donor patient was used as a source of antibodies within 28 days of collection, and several human trials are being conducted to better understand and evaluate CP as a method of treatment for COVID-19 [92]. Currently, The FDA only advises the use of COVID-19 CP under emergency use authorization (EUA) or investigational CP under an investigational new drug (IND) during a public health emergency [93].

Currently, there is no evidence to recommend that any specific COVID-19 treatment exists; however, several drugs and potential therapeutic strategies that target different parts of the SARS-CoV-2 life cycle in addition to host biology are under investigation, and many clinical trials are being registered and updated at https://clinicaltrials.gov/ [94].

## 4. Nano-Biomimetic Drug Delivery Technologies as Potential Treatment Strategies for COVID-19

Nanotechnology has the potential to facilitate the development of diverse drug delivery systems for the treatment of COVID-19 infections primarily due to their small size, morphology, and ability to mimic human cell or cellular component behavior. A wide range of APIs, including antiviral, biologic, and nucleic acid compounds, can be loaded into and delivered by nanocarriers. This approach facilitates selection of an appropriate nanocarrier and therapeutic agent for a specific disease condition and is vital if commercial success of a nanomedicine for the SARS-CoV-2 virus is to be achieved [95]. Biomimicry permits use of naturally-derived cell components such as membranes, for example, and make use of multivalent cell membrane markers simultaneously ranging from targeting to immunomodulation of cell surface markers [96]. These features allow biomimetic nanoparticles (NP) to target and reach physiologically inaccessible sites whilst eliminating the immune response of the reticular endothelial cells while potentially offering alternative treatment targets in vulnerable cells in COVID-19 infections. This concept forms the basis for evaluation and the potential application of nano biomimetics in COVID-19 theranostics.

### 4.1. Nano Macrophage-Mimetic Drug Delivery for COVID-19

Macrophages have a number of roles in human biology, including development of tissues, homeostasis, repair, and more specifically innate immunity, which is pertinent to this review [97]. Nano macrophage-mimetic (NMM) drug delivery is influenced by the identification and selection of receptors located on the surface of macrophages and the enhancement of the nanomedicines towards these receptors [56]. Macrophage-inspired targeted drug delivery has been explored for the treatment of lung cancer [98].

Macrophage-mimetic nanoparticles (MMNPs) have an antigenic exterior surface, which is similar or the same as human macrophage cells, and are able to bind to endotoxins. Therefore, MMNPs can act as a decoy to organisms such as bacteria and viruses, ensuring management and prevention of infection is possible [99,100,101].

The use of an MMNP for treatment in a murine *Escherichia coli* bacteremia model revealed a reduction in pro-inflammatory cytokine levels and inhibition of bacterial dissemination, thereby guaranteeing survival of the infected animals [102].

Cellular nanosponges produced from human cell membranes have been used as a medical countermeasure to COVID-19 infection [101]. Macrophages were attached to the surface of the nanosponges and mimicked ACE-2 and CD147 target receptors, and this was verified using Western blot analysis [101]. A dose-dependent inhibition of the viral infectivity was observed [101].

In principle, as long as the virus target remains in the identified host cell, the MMNP can neutralize the infection by providing broad-acting coverage. These nano macrophage-mimetic systems can neutralize viral activity during the initial stages of COVID-19 infections and decrease the fulminant inflammation associated with COVID-19 in the later stages of the disease [101]. MMNPs have broad-spectrum neutralization capabilities and exhibit activity against bacterial toxins and inflammatory cytokines [102]. However, since nanoparticles are able to trigger internal inflammation whilst targeting host cells, the particle size and surface coating on the particles are critical parameters that must be investigated during the development of MMNPs and should range in size between 120 and 200 nm to prevent inflammation [103].

### 4.2. Nano Erythrocyte-Mimetic Drug Delivery for COVID-19

The desirable properties of erythrocyte-mimetic technologies as drug delivery vehicles are based on their structure and the surface proteins used. It is possible to exploit these properties using these as design cues to design and develop next-generation nano biomimetic delivery platforms [104,105,106]. Despite significant research activity to narrow the gap between synthetic nanomaterials and biological entities, a erythrocyte-mimicking delivery vehicle remains elusive due, in part, to the challenge of functionalizing NP so as to mimic the complex surface chemistry of biological cell in vivo [104].

Type II and III erythrocytes have been most commonly evaluated for drug delivery and, by way of example, a combination of photothermal effect enhanced hollow mesoporous Prussian blue (HMPB) NP with an erythrocyte membrane camouflage and folic acid modifications has been demonstrated successfully [107]. The nanoplatform developed was precise, exhibited controlled release and sustained accumulation of doxorubicin (DOX) whilst demonstrating a high drug loading capacity due to the large surface area and pore volume [107].

Similarly, erythrocyte-mimetic nanoparticles (EMNP) were developed for the treatment of paraoxon toxicity [108] and cancers with paclitaxel (PTX) [109] and DOX [20].

Type III EMNP were developed for delivering PTX to lung tumors and significantly enhanced perfusion into the primary tumor and, more specifically, lung metastases when co-administered with a tumor-penetrating peptide iRGD [109]. These findings provide novel approaches for the design of nanocarriers intended to target delivery of therapeutic compounds to tumors.

Type III EMNPs intended for the delivery of DOX were synthesized using physical encapsulation or chemical conjugation [20], and release studies suggested that chemical conjugation resulted in a longer duration of sustained release of DOX than physical encapsulation.

It is generally believed the S glycoprotein located on surface of the SARS-CoV-2 virus enters erythrocytes and binds to β chains of hemoglobin and, in some instances, interferes directly with the heme functionality of the molecule with the ultimate effect of binding methemoglobinemia [110,111]. The subsequent reduction in the production of hemoglobin that occurs is a consequence of oxidative stress. Hyperferritinemia and a reduction of the T4 helper cell population coupled with the production of reactive oxygen (ROS) and reactive nitrogen species (RNS) ensues [112]. Notwithstanding the role of immune-inflammation processes in the pathophysiology of COVID-19, hemoglobin alteration, hypoxemia, and iron dysmetabolism represent additional factors to be investigated as theranostic targets to consider type II and III EMNP as potential drug delivery tools.

EMNPs have the potential to combine important drug delivery properties such as biocompatibility, colloidal stability, and long circulatory retention times, and a deeper understanding of the role played by the erythrocyte shell and polymeric core may permit further engineered modification of these nano-formulations to subsequently improve the systemic delivery of potential therapeutic payloads. Furthermore, the use of EMNP may permit development of decoy targets for the SARS-Cov-2 virus and subsequently reduce the effects of some of the hematological pathology of COVID-19 infections [113,114].

### 4.3. Nano Platelet-Mimetic Drug Delivery for COVID-19

Reports of thrombocytopenia, pulmonary vascular leakage, thrombi, and disseminated intravascular coagulation (DIC) in COVID-19 patients are common and are associated with an increase in morbidity and mortality rates [115,116]. Thrombocytopenia observed in patients may be a result of either an immune response mediated thrombocytosis leading to immune thrombocytopenia (ITP) [116], or it may be a side effect of drugs such as heparin, azithromycin, and hydrochloroquine used to treat COVID-19 patients [115,117]. The host organism regulates platelet production in order to minimize the inflammatory storm and beneficial platelet–pathogen interactions, which protect pathogens from identification by the immune cells and cytotoxic agents [116]. Three main mechanisms of SARS-CoV-2 induced thrombocytopenia have been proposed: decreased platelet synthesis via direct infection of the bone marrow and trauma to the lungs, increased destruction of platelets due to an immune response, and increased consumption of platelets in the lungs [116,118,119]. The decrease in platelet synthesis via direct infection of the bone marrow by SARS-CoV-2 results in inhibition of hematopoiesis and blockage of platelet release from pulmonary megakaryocytes following trauma to the lung tissue. Autoantibody and immune complexes produced following a SARS-CoV-2 infection are deposited onto the surfaces of platelets, which are targeted for destruction by the host immune system. Lastly, damage to the lungs following a SARS-CoV-2 infection results in increased consumption of platelets, as they aggregate at the site of injury and form thrombi lungs [116,118,119].

The interaction between platelets and pathogens shields pathogens from an immune response by host organisms [120], and this challenge can be overcome by use of platelet-mimetic nanoparticle (PMNP) technology to treat infections. PMNP technology makes use of platelet membranes to disguise API-containing nanoparticles and decrease clearance of such particles, which would otherwise be regarded as antigenic [120]. API-containing silica or poly (lactic-co-glycolic acid) (PLGA) polymeric nanoparticles, incorporated into platelet membranes isolated from whole blood by centrifugation, are functionalized with a specific receptor for a target pathogen [120]. The target pathogen binds to the specific receptors on the platelet, and the API-containing composite enters the infecting virus and destroys it [120,121]. Specific receptors can be attached to platelet mimetics to ensure death of pathogens [120], and this approach has been investigated for the treatment of cancer [122,123], bacterial infection [124], and vascular damage [125].

Polymeric nanoparticles have also been used for the treatment of a variety of diseases and conditions, but generally exhibit short in-vivo circulation times and are non-specific and incompatible with biological tissues, thereby triggering immune responses [123]. Surface modifiers and specific proteins, when added to nanoparticles, can improve recognition by target cells; however, if these do not match endogenous compounds, the result is removal of the nanoparticles via an immune response [123]. The use of a PMNP ensures longer residence time for the payload in-vivo, thereby enhancing therapeutic outcomes by providing a specific target for the platelet-binding pathogen, and by shielding the technology from destruction by macrophage phagocytosis due to the presence of specific membrane proteins [120,123]. The usual platelet induced inflammatory response is eliminated when using platelet membranes alone as opposed to whole platelets [126]. The morphology, flexibility, and ability of platelets to aggregate and recruit additional activated platelets in order to perform their function at the site of vascular injury makes PMNP useful in managing COVID-19 induced thrombocytopenia and vascular damage [125].

### 4.4. Nano Virus-Mimetic Drug Delivery for COVID-19

Viruses can efficiently bind to host cells by specific interactions between virion proteins and membrane lipids, proteins, and/or carbohydrate moieties on the surface of the cells. Following attachment, virus entry into the host cells occurs via endocytosis/pinocytosis or fusion/penetration. Furthermore viruses have developed strategies to evade the immune system of the host, and different approaches have been established to construct biomimetic nanoparticles to take advantage of the unique capabilities of viruses to adapt and evade recognition [127].

Four types of virus-mimetic nanoparticles, viz., virosomes, virus-like particles (VP), self-assembling nanoparticles with surface antigens, and fully synthetic virus mimicking nanoparticle have been described [127].

The use of virosomes entails incorporation of virus-derived proteins in lamellar spherical liposomes consisting of phospholipid bilayers and ranging in size between 20 and 200 nm [128,129]. In general the enveloped glycoproteins derived from influenza virus, such as hemagglutinin (HA) and neuraminidase (NA), are reconstituted with liposomes to prepare virosomes for vaccination or delivery of different therapeutic agents [130]. Furthermore, other enveloped viruses, such as hemagglutinating virus of Japan (HVJ), respiratory syncytial virus (RSV), and vesicular stomatitis virus (VSV), can be used to prepare virosomes [131,132,133,134]. In other cases, human hepatitis B virus-derived nanoparticles have been fused with liposomes, giving rise to virosome-like particles [131,134,135]. The lipoprotein inclusion results in structural stability of the virosomes and is responsible for disease targeting, cellular uptake, and endolysosomal escape following internalization of the carrier. Virosomes exhibit a number of advantages over other technologies including ease of production and modification, biodegradability, biocompatibility, and promotion of fusion activity in the endolysosomes, whilst permitting the delivery of different drugs and protecting biologics such as monoclonal antibodies (MAb) from degradation [136]. Nevertheless, their broad application remains limited, largely due to the potential risk of immunogenicity which can be partly addressed by the modification of the virosome surface with polyethylene glycol (PEG) and/or ligands [137,138], including antibodies, in order to reduce off-target effects [137,139].

Virus-like particles (VLPs) are assembled using viral capsids or envelope proteins derived from viruses, and these precisely defined structures enhance the loading capacity and packaging of different drugs whilst displaying functional moieties on their surfaces, and, importantly, VLPs can also be formed using synthetic viral capsids [140]. Pristine VLPs can be further modified to ensure additional functionality by tailoring VLP proteins via genetic and chemical engineering [141,142], such as, for instance, conjugation of hydrophilic polymers to the VLP to increase stability, prolong circulation time, reduce non-specific adsorption, or attenuate immune responses [143,144]. To overcome the disadvantages of the natural tropism of VLPs, different chemical functionalization approaches have been developed to conjugate different ligands on VLPs for site-specific targeting and drug delivery [142]. Since the antigenicity of VLPs is comparable to that of the original virus, they were initially used for vaccination [145]. VLPs for MERS-CoV (MERS-CoV-LP) have been developed via co-expression of S, E, and M proteins in Bm5 cells and the consequent self-assembly of S protein-displaying NP in the 100–200 nm size range from cultured cells by mechanical extrusion [146]. A slight modification of these NPs with SARS-CoV-2 S protein permits NP attachment to ACE-2 receptors instead of dipeptidyl-peptidase 4 (DPP4), resulting in stimulation of the immune system [147]. Another self-assembling approach for MERS-CoV-RBD fused with VP2 structural protein gene of canine parvovirus in insect cells is to produce RBD-displaying chimeric VLPs of approximately 50 nm in size, which were able to express the RBD [148]. VLP can be engineered to deliver different drugs including small-molecules, peptides, protein and nucleic acids where the therapeutic molecules are retained by non-covalent interaction-mediated physical loading or chemical conjugation [149,150].

Self-assembling nanoparticles are produced by use of viral glycoproteins and natural proteins that have the ability to form nanoparticles spontaneously, as observed with influenza HA when genetically fused to ferritin, where the resultant fusion glycoprotein formed nanoparticles spontaneously whilst exposing eight HA trimers on the surface [149]. Recently, a computational protein design approach was used to develop a self-assembling nanoparticle bearing an RSV antigen [151]. In this case, a rationally designed, self-assembling protein nanoparticle served as a scaffold for multivalent presentation of a prefusion-stabilized variant of the F glycoprotein trimer of RSV, with a repetitive array and controllable density, and the in silico designed and fully synthetic nanoparticle exhibited optimal stability and limited immunogenicity [151].

Nanovaccines are fabricated by encapsulation of the CoV antigens or exposing the antigen on the surface of the NP, thereby producing NPs of similar immunological conformation to the virus. The S protein is the main attachment factor and immunodominant antigen in the CoV and is therefore a prime candidate for nanovaccine development [147], indicating that structure-based assembly is the commonly used method for the production of coronaviral nanovaccines [147].

The S protein trimers can be self-assembled by removal of a non-ionic surfactant during the purification process when forming the NPs, and mice vaccinated with NPs synthesized for use against SARS-CoV induced a high level of neutralizing antibodies, which increased 15-fold and 68-fold when aluminum hydroxide and Matrix M1 were used as adjuvants, respectively [152].

## 5. Conclusions

The COVID-19 pandemic continues to be a global catastrophe with positive cases rapidly increasing in number throughout the world. Consequently, the development of conventional drugs, medicines, and vaccines, in addition to the use of novel drug delivery technologies, has gained momentum in the fight against this pandemic. State of the art delivery technologies, such as the use of nanospheres/nanocapsules, nanocrystals, liposomes, solid lipid nanoparticles/nano lipid carriers, dendrimers, and nanosponges, based on biomimicry, can be harnessed for targeted delivery of therapeutic compounds to infected individuals for the treatment of COVID-19. However, the expansions of knowledge and understanding of the COVID-19 pandemic are emerging daily, necessitating the use of flexible and agile strategies to curb the ongoing spread of the virus. While researchers continue to seek treatment and/or vaccine development strategies, there is a need to continue to use existing non-pharmacological interventions to prevent the spread of infection, which include but are not limited to regular cleaning and disinfection of surfaces, handwashing and sanitization, physical distancing, wearing a mask, and imposing travel restrictions.

## Figures and Tables

**Table 1:** Staging and clinical features of COVID-19 [32].

Clinical Staging	Clinical Manifestation
Asymptomatic	None
Mild	Acute upper respiratory tract infection with symptoms such as fever, fatigue, myalgia, dry cough, sore throat, runny nose, sneezing, and/or digestive symptoms presenting as nausea, vomiting, abdominal pain, and diarrhea
Moderate	Pneumonia with frequent fever, cough with no obvious hypoxemia, chest CT with lesions (ground glass appearance)
Severe	Pneumonia with hypoxemia (oxygen saturation < 92%)
Critical	ARDS, acute renal damage, possibly shock, encephalopathy, myocardial damage, heart failure, and coagulation dysfunction

## References

[1] Pandey A., Nikam A.N., Shreya A.B., Mutalik S.P., Gopalan D., Kulkarni S., Padya B.S., Fernandes G., Mutalik S., Prassl R. (2020). Potential therapeutic targets for combating SARS-CoV-2: Drug repurposing, clinical trials and recent advancements. Life Sci..

[2] Huang C., Wang Y., Li X., Ren L., Zhao J., Hu Y., Zhang L., Fan G., Xu J., Gu X. (2020). Clinical features of patients infected with 2019 novel coronavirus in Wuhan, China. Lancet.

[3] Ksiazek T.G., Erdman D., Goldsmith C.S., Zaki S.R., Peret T., Emery S., Tong S., Urbani C., Comer J.A., Lim W. (2003). A novel coronavirus associated with severe acute respiratory syndrome. N. Engl. J. Med..

[4] Zhang J., Litvinova M., Wang W., Wang Y., Deng X., Chen X., Li M., Zheng W., Yi L., Chen X. (2020). Evolving epidemiology and transmission dynamics of coronavirus disease 2019 outside Hubei province, China: A descriptive and modelling study. Lancet Infect. Dis..

[5] Johns Hopkins University Coronavirus Resource Center. https://coronavirus.jhu.edu/map.html.

[6] Zhang H., Penninger J.M., Li Y., Zhong N., Slutsky A.S. (2020). Angiotensin-converting enzyme 2 (ACE2) as a SARS-CoV-2 receptor: Molecular mechanisms and potential therapeutic target. Int. Care Med..

[7] Shi H., Han X., Jiang N., Cao Y., Alwalid O., Gu J., Fan Y., Zheng C. (2020). Radiological findings from 81 patients with COVID-19 pneumonia in Wuhan, China: A descriptive study. Lancet Infect. Dis..

[8] Coperchini F., Chiovato L., Croce L., Magri F., Rotondi M. (2020). The cytokine storm in COVID-19: An overview of the involvement of the chemokine/chemokine-receptor system. Cytokine Growth Factor Rev..

[9] National Institutes of Health (NIH) Clinical Presentation, COVID-19 Treatment Guidelines. https://www.covid19treatmentguidelines.nih.gov/overview/clinical-presentation.

[10] Boulware D.R., Pullen M.F., Bangdiwala A.S., Pastick K.A., Lofgren S.M., Okafor E.C., Skipper C.P., Nascene A.A., Nicol M.R., Abassi M. (2020). A Randomized trial of hydroxychloroquine as postexposure prophylaxis for Covid-19. N. Engl. J. Med..

[11] Gautret P., Lagier J.C., Parola P., Hoang V.T., Meddeb L., Mailhe M., Doudier B., Courjon J., Giordanengo V., Vieira V.E. (2020). Hydroxychloroquine and azithromycin as a treatment of COVID-19: Results of an open-label non-randomized clinical trial. Int. J. Antimicrob. Agents.

[12] Cavalcanti A.B., Zampieri F.G., Rosa R.G., Azevedo L.C.P., Veiga V.C., Avezum A., Damiani L.P., Marcadenti A., Kawano-Dourado L., Lisboa T. (2020). Hydroxychloroquine with or without azithromycin in mild-to-moderate Covid-19. N. Engl. J. Med..

[13] Cao B., Wang Y., Wen D., Liu W., Wang J., Fan G., Ruan L., Song B., Cai Y., Wei M. (2020). A Trial of lopinavir–ritonavir in adults hospitalized with severe Covid-19. N. Engl. J. Med..

[14] Beigel J.H., Tomashek K.M., Dodd L.E., Mehta A.K., Zingman B.S., Kalil A.C., Hohmann E., Chu H.Y., Luetkemeyer A., Kline S. (2020). Remdesivir for the treatment of COVID-19—Preliminary report. N. Engl. J. Med..

[15] Patra J.K., Das G., Fraceto L.F., Vangelie E., Campos R., Rodriguez-Torres M.D.P., Acosta-Torres L.S., Diaz-Torres L.A., Grillo R., Swamy M.K. (2018). Nano based drug delivery systems: Recent developments and future prospects. J. Nanobiotechnol..

[16] Chen Z., Wang Z., Gu Z. (2019). Bioinspired and biomimetic nanomedicines. Acc. Chem. Res..

[17] Witika B.A., Makoni P.A., Matafwali S.K., Chabalenge B., Mwila C., Kalungia A.C., Nkanga C.I., Bapolisi A.M., Walker R.B. (2020). Biocompatibility of biomaterials for nanoencapsulation: Current approaches. Nanomaterials.

[18] Sheikhpour M., Barani L., Kasaeian A. (2017). Biomimetics in drug delivery systems: A critical review. J. Control. Release.

[19] Jiang L., Li R., Xu J., Luan P., Cui Q., Pang Z., Wang J., Lin G., Zhang J. (2019). Endotoxin-adsorbing macrophage-mimetic hybrid liposome for sepsis treatment. Chem. Eng. J..

[20] Aryal S., Hu C.M.J., Fang R.H., Dehaini D., Carpenter C., Zhang D.E., Zhang L. (2013). Erythrocyte membrane-cloaked polymeric nanoparticles for controlled drug loading and release. Nanomedicine.

[21] Doshi N., Orje J.N., Molins B., Smith J.W., Mitragotri S., Ruggeri Z.M. (2012). Platelet mimetic particles for targeting thrombi in flowing blood. Adv. Mater..

[22] Gupta S., Krishnakumar V., Sharma Y., Dinda A.K., Mohanty S. (2020). Mesenchymal stem cell derived exosomes: A nano platform for therapeutics and drug delivery in combating COVID-19. Stem Cell Rev. Rep..

[23] Somiya M., Kuroda S. (2015). Development of a virus-mimicking nanocarrier for drug delivery systems: The bio-nanocapsule. Adv. Drug Deliv. Rev..

[24] Zhang P., Chen Y., Zeng Y., Shen C., Li R., Guo Z., Li S., Zheng Q., Chu C., Wang Z. (2015). Virus-mimetic nanovesicles as a versatile antigen-delivery system. Proc. Natl. Acad. Sci. USA.

[25] Lang T., Yin Q., Li Y. (2018). Progress of cell-derived biomimetic drug delivery systems for cancer therapy. Adv. Ther..

[26] Nowacek A.S., Mcmillan J., Miller R., Anderson A., Rabinow B., Gendelman H.E. (2012). Macrophages: Implications for neuroAIDS therapeutics. J. Neuroimmune Pharmacol..

[27] Nowacek A.S., Miller R.L., Mcmillan J., Kanmogne G., Mosley R.L., Ma Z., Graham S., Chaubal M., Rabinow B., Dou H. (2009). NanoART synthesis, characterization, uptake, release and toxicology for human monocyte—macrophage drug delivery. Nanomedicine.

[28] Witika B.A., Smith V.J., Walker R.B. (2020). Quality by design optimization of cold sonochemical synthesis of zidovudine-lamivudine nanosuspensions. Pharmaceutics.

[29] Papadia K., Giannou A.D., Markoutsa E., Bigot C., Vanhoute G., Mourtas S., Van der Linded A., Stathopoulos G.T., Antimisiaris S.G. (2017). Multifunctional LUV liposomes decorated for BBB and amyloid targeting—B. In vivo brain targeting potential in wild-type and APP/PS1 mice. Eur. J. Pharm. Sci..

[30] Makhlof A., Tozuka Y., Takeuchi H. (2009). pH-Sensitive nanospheres for colon-specific drug delivery in experimentally induced colitis rat model. Eur. J. Pharm. Biopharm..

[31] Channappanavar R., Zhao J., Perlman S. (2014). T cell-mediated immune response to respiratory coronaviruses. Immunol. Res..

[32] Yuki K., Fujiogi M., Koutsogiannaki S. (2020). COVID-19 pathophysiology: A review. Clin. Immunol..

[33] Mason R.J. (2020). Pathogenesis of COVID-19 from a cell biology perspective. Eur. Respir. J..

[34] Matheson B.N.J., Lehner P.J. (2020). How does SARS-CoV-2 cause COVID-19?. Science.

[35] Rabi F.A., Al Zoubi M.S., Al-Nasser A.D., Kasasbeh G.A., Salameh D.M. (2020). SARS-CoV-2 and coronavirus disease 2019: What we know so far. Pathogens.

[36] Perlman S., Netland J. (2009). Coronaviruses post-SARS: Update on replication and pathogenesis. Nat. Rev. Microbiol..

[37] Li F. (2016). Structure, function, and evolution of coronavirus spike proteins. Annu. Rev. Virol..

[38] Du L., Yang Y., Zhou Y., Lu L., Li F., Jiang S. (2017). MERS-CoV spike protein: A key target for antivirals. Expert Opin. Ther. Targets.

[39] Du L., He Y., Zhou Y., Liu S., Zheng B.J., Jiang S. (2009). The spike protein of SARS-CoV—A target for vaccine and therapeutic development. Nat. Rev. Microbiol..

[40] Ciulla M.M. (2020). Coronavirus uses as binding site in humans angiotensin-converting enzyme 2 functional receptor that is involved in arterial blood pressure control and fibrotic response to damage and is a drug target in cardiovascular disease. Is this just a phylogenetic. J. Med. Virol..

[41] Li F. (2015). Receptor recognition mechanisms of coronaviruses: A decade of structural studies. J. Virol..

[42] Zhao Y., Zhao Z., Wang Y., Zhou Y., Ma Y., Zuo W. (2020). Single-cell RNA expression profiling of ACE2, the receptor of SARS-CoV-2. bioRxiv.

[43] Kuba K., Imai Y., Rao S., Jiang C., Penninger J.M. (2006). Lessons from SARS: Control of acute lung failure by the SARS receptor ACE2. J. Mol. Med..

[44] Hussman J.P. (2020). Cellular and molecular pathways of COVID-19 and potential points of therapeutic intervention. Front. Pharmacol..

[45] Andersen K.G., Rambaut A., Lipkin W.I., Holmes E.C., Garry R.F. (2020). The proximal origin of SARS-CoV-2. Nat. Med..

[46] Ouassou H., Kharchoufa L., Bouhrim M., Daoudi N.E., Imtara H., Bencheikh N., Elbouzidi A., Bnouham M. (2020). The Pathogenesis of coronavirus disease 2019 (COVID-19): Evaluation and prevention. J. Immunol. Res..

[47] Yao X.H., He Z.C., Li T.Y., Zhang H.R., Wang Y., Mou H., Guo Q., Yu S.C., Ding Y., Liu X. (2020). Pathological evidence for residual SARS-CoV-2 in pulmonary tissues of a ready-for-discharge patient. Cell Res..

[48] Wu J., Wu X., Zeng W., Guo D., Fang Z., Chen L., Huang H., Li C. (2020). Chest CT findings in patients with coronavirus disease 2019 and its relationship with clinical features. Invest. Radiol..

[49] Sterne J.A.C., Murthy S., Diaz J.V., Slutsky A.S., Villar J., Angus D.C., Annane D., Azevedo L.C.P., Berwanger O., Cavalcanti A.B. (2020). Association between administration of systemic corticosteroids and mortality among critically ILL patients with COVID-19: A meta-analysis. JAMA.

[50] Sternberg A., McKee D.L., Naujokat C. (2020). Novel drugs targeting the SARS-CoV-2/COVID-19 machinery. Curr. Top. Med. Chem..

[51] Kruse R.L. (2020). Therapeutic strategies in an outbreak scenario to treat the novel coronavirus originating in Wuhan, China. F1000Research.

[52] Cao Y., Li L., Feng Z., Wan S., Huang P., Sun X., Wen F., Huang X., Ning G., Wang W. (2020). Comparative genetic analysis of the novel coronavirus (2019-nCoV/SARS-CoV-2) receptor ACE2 in different populations. Cell Discov..

[53] Letko M., Marzi A., Munster V. (2020). Functional assessment of cell entry and receptor usage for SARS-CoV-2 and other lineage B betacoronaviruses. Nat. Microbiol..

[54] Khattabi L. (2020). Recombinant protein targeting and opsonizing spike glycoprotein for enhancing SARS-CoV-2 phagocytosis. Med. Hypotheses.

[55] Murin C.D., Wilson I.A., Ward A.B. (2019). Antibody responses to viral infections: A structural perspective across three different enveloped viruses. Nat. Microbiol..

[56] Lei C., Fu W., Qian K., Li T., Zhang S., Ding M., Hu S. (2020). Potent neutralization of 2019 novel coronavirus by recombinant ACE2-Ig. bioRxiv.

[57] Monteil V., Kwon H., Prado P., Hagelkrüys A., Wimmer R.A., Stahl M., Leopoldi A., Garreta E., Pozo C.H.D., Prosper F. (2020). Inhibition of SARS-CoV-2 infections in engineered human tissues using clinical-grade soluble human ACE2. Cell.

[58] Walls A.C., Park Y.-J., Tortorici M.A., Wall A., McGuire A.T., Veesler D. (2020). Structure, function, and antigenicity of the SARS-CoV-2 spike glycoprotein. Cell.

[59] Zhang G., Pomplun S., Loftis A.R., Loas A., Pentelute B.L. (2020). The first-in-class peptide binder to the SARS-CoV-2 spike protein. bioRxiv.

[60] Iwata-Yoshikawa N., Okamura T., Shimizu Y., Hasegawa H., Takeda M., Nagata N. (2019). TMPRSS2 contributes to virus spread and immunopathology in the airways of murine models after coronavirus infection. J. Virol..

[61] Hoffmann M., Kleine-Weber H., Schroeder S., Krüger N., Herrler T., Erichsen S., Schiergens T.S., Herrler G., Wu N.H., Nitsche A. (2020). SARS-CoV-2 cell entry depends on ACE2 and TMPRSS2 and is blocked by a clinically proven protease inhibitor. Cell.

[62] Kawase M., Shirato K., Van der Hoek L., Taguchi F., Matsuyama S. (2020). Simultaneous treatment of human bronchial epithelial cells with serine and cysteine protease inhibitors prevents severe acute respiratory syndrome coronavirus entry. J. Virol..

[63] Rahman N., Basharat Z., Yousuf M., Castaldo G., Rastrelli L., Khan H. (2020). Virtual screening of natural products against type II transmembrane serine protease (TMPRSS2), the priming agent of coronavirus 2 (SARS-COV-2). Molecules.

[64] Hoffmann M., Schroeder S., Kleine-Weber H., Müller M.A., Drosten C., Pöhlmann S. (2020). Nafamostat mesylate blocks activation of SARS-CoV-2: New treatment option for COVID-19. Antimicrob. Agents Chemother..

[65] Qing E., Hantak M., Perlman S., Gallagher T. (2020). Distinct roles for sialoside and protein receptors in coronavirus infection. MBio.

[66] Vankadari N. (2020). Structure of furin protease binding to SARS-CoV-2 spike glycoprotein and implications for potential targets and virulence. J. Phys. Chem. Lett..

[67] Cheng Y.W., Chao T.L., Li C.L., Chiu M.F., Kao H.C., Wang S.H., Pang Y.H., Lin C.H., Tsai Y.M., Lee W.H. (2020). Furin inhibitors block SARS-CoV-2 Spike protein cleavage to suppress virus production and cytopathic effects. Cell Rep..

[68] Xia S., Liu M., Wang C., Xu W., Lan Q., Feng S., Qi F., Bao L., Du L., Liu S. (2020). Inhibition of SARS-CoV-2 (previously 2019-nCoV) infection by a highly potent pan-coronavirus fusion inhibitor targeting its spike protein that harbors a high capacity to mediate membrane fusion. Cell Res..

[69] Yang N., Shen H.M. (2020). Targeting the endocytic pathway and autophagy process as a novel therapeutic strategy in COVID-19. Int. J. Biol. Sci..

[70] Oscanoa T.J., Romero-Ortuno R., Carvajal A., Savarino A. (2020). A pharmacological perspective of chloroquine in SARS-CoV-2 infection: An old drug for the fight against a new coronavirus?. Int. J. Antimicrob. Agents.

[71] Amin M., Abbas G. (2020). Docking study of chloroquine and hydroxychloroquine interaction with RNA binding domain of nucleocapsid phospho-protein—An in silico insight into the comparative efficacy of repurposing antiviral drugs. J. Biomol. Struct. Dyn..

[72] Devaux C.A., Rolain J.M., Colson P., Raoult D. (2020). New insights on the antiviral effects of chloroquine against coronavirus: What to expect for COVID-19?. Int. J. Antimicrob. Agents.

[73] Khuroo M.S. (2020). Chloroquine and hydroxychloroquine in coronavirus disease 2019 (COVID-19). Facts, fiction and the hype: A critical appraisal. Int. J. Antimicrob. Agents.

[74] Horby P., Mafham M., Linsell L., Bell J.L., Staplin N., Emberson J.R., Wiselka M., Ustianowski A., Elmahi E., Prudon B. (2020). Hydroxychloroquine for COVID-19-preliminary report effect of hydroxychloroquine in hospitalized patients. medRxiv.

[75] Hukowska-Szematowicz B. (2020). Genetic variability and phylogenetic analysis of Lagovirus europaeus strains GI.1 (RHDV) and GI.2 (RHDV2) based on the RNA-dependent RNA polymerase (RdRp) coding gene. Acta Biochimica Polonica.

[76] Xu X., Liu Y., Weiss S., Arnold E., Sarafianos S.G., Ding J. (2003). Molecular model of SARS coronavirus polymerase: Implications for biochemical functions and drug design. Nucleic Acids Res..

[77] Wang M., Cao R., Zhang L., Yang X., Liu J., Xu M., Shi Z., Hu Z., Zhong W., Xiao G. (2020). Remdesivir and chloroquine effectively inhibit the recently emerged novel coronavirus (2019-nCoV) in vitro. Cell Res..

[78] Furuta Y., Komeno T., Nakamura T. (2017). Favipiravir (T-705), a broad spectrum inhibitor of viral RNA polymerase. Proc. Jpn. Acad. Ser. B Phys. Biol. Sci..

[79] Cai Q., Yang M., Liu D., Chen J., Shu D., Xia J., Liao X., Gu Y., Cai Q., Yang Y. (2020). Experimental treatment with Favipiravir for COVID-19: An open-label control study. Engineering.

[80] Saber-Ayad M., Saleh M.A., Abu-Gharbieh E. (2020). The rationale for potential pharmacotherapy of covid-19. Pharmaceuticals.

[81] Cheng J.L., Huang C., Zhang G.J., Liu D.W., Li P., Lu C.Y., Li J. (2020). Epidemiological characteristics of novel coronavirus pneumonia in Henan. Zhonghua Jie He He Hu Xi Za Zhi.

[82] Eastman R.T., Roth J.S., Brimacombe K.R., Simeonov A., Shen M., Patnaik S., Hall M.D. (2020). Remdesivir: A review of its discovery and development leading to emergency use authorization for treatment of COVID-19. ACS Cent. Sci..

[83] Zheng M., Williams E.P., Malireddi R.K.S., Karki R., Banoth B., Burton A., Webby R., Channappanavar R., Jonsson C.B., Kanneganti T.D. (2020). Impaired NLRP3 inflammasome activation/pyroptosis leads to robust inflammatory cell death via caspase-8/RIPK3 during coronavirus infection. J. Biol. Chem..

[84] Mehta P., McAuley D.F., Brown M., Sanchez E., Tattersall R.S., Manson J.J. (2020). COVID-19: Consider cytokine storm syndromes and immunosuppression. Lancet.

[85] Bonaventura A., Vecchié A., Wang T.S., Lee E., Cremer P.C., Carey B., Rajendram P., Hudock K.M., Korbee L., Van Tassell B.W. (2020). Targeting GM-CSF in COVID-19 pneumonia: Rationale and strategies. Front. Immunol..

[86] Chen Z., John Wherry E. (2020). T cell responses in patients with COVID-19. Nat. Rev. Immunol..

[87] Veronese N., Demurtas J., Yang L., Tonelli R., Barbagallo M., Lopalco P., Lagolio E., Celotto S., Pizzol D., Zou L. (2020). Use of corticosteroids in coronavirus disease 2019 pneumonia: A systematic review of the literature. Front. Med..

[88] Horby P., Lim W.S., Emberson J., Mafham M., Bell J., Linsell L., Staplin N., Brightling C., Ustianowski A., Elmahi E. (2020). Effect of dexamethasone in hospitalized patients with COVID-19: Preliminary report. medRxiv.

[89] Zhang C., Wu Z., Li J.W., Zhao H., Wang G.Q. (2020). Cytokine release syndrome in severe COVID-19: Interleukin-6 receptor antagonist tocilizumab may be the key to reduce mortality. Int. J. Antimicrob. Agents.

[90] Focosi D., Anderson A.O., Tang J.W., Tuccori M. (2020). Convalescent plasma therapy for COVID-19: State of the art. Clin. Microbiol. Rev..

[91] Mair-Jenkins J., Saavedra-Campos M., Baillie J.K., Cleary P., Khaw F.M., Lim W.S., Makki S., Rooney K.D., Nguyen-Van-Tam J.S., Beck C.R. (2015). The effectiveness of convalescent plasma and hyperimmune immunoglobulin for the treatment of severe acute respiratory infections of viral etiology: A systematic review and exploratory meta-analysis. J. Infect. Dis..

[92] Salazar E., Christensen P.A., Graviss E.A., Nguyen D.T., Castillo B., Chen J., Lopez B.V., Eagar T.N., Yi X., Zhao P. (2020). Treatment of COVID-19 patients with convalescent plasma reveals a signal of significantly decreased mortality. Am. J. Pathol..

[93] FDA Recommendations for Investigational COVID-19 Convalescent Plasma. https://www.fda.gov/vaccines-blood-biologics/investigational-new-drug-ind-or-device-exemption-ide-process-cber/recommendations-investigational-covid-19-convalescent-plasma.

[94] US National Library of Medicine COVID-19—Clinical Trials. https://clinicaltrials.gov/ct2/results?cond=COVID-19.

[95] Chauhan G., Madou M.J., Kalra S., Chopra V., Ghosh D., Martinez-Chapa S.O. (2020). Nanotechnology for COVID-19: Therapeutics and vaccine research. ACS Nano.

[96] Yoo J.W., Irvine D.J., Discher D.E., Mitragotri S. (2011). Bio-inspired, bioengineered and biomimetic drug delivery carriers. Nat. Rev. Drug Discov..

[97] Wynn T.A., Chawla A., Pollard J.W. (2013). Origins and hallmarks of macrophages: Development, homeostasis, and disease. Nature.

[98] Cao H., Dan Z., He X., Zhang Z., Yu H., Yin Q., Li Y. (2016). Liposomes coated with isolated macrophage membrane can target lung metastasis of breast cancer. ACS Nano.

[99] Wei X., Zhang G.C., Ran D., Krishnan N., Fang R.H., Gao W., Spector S.A., Zhang L. (2018). T-cell-mimicking nanoparticles can neutralize HIV infectivity. Adv. Mater..

[100] Dehaini D., Fang R.H., Zhang L. (2016). Biomimetic strategies for targeted nanoparticle delivery. Bioeng. Transl. Med..

[101] Zhang Q., Honko A., Zhou J., Gong H., Downs S.N., Vasquez J.H., Fang R.H., Gao W., Griffiths A., Zhang L. (2020). Cellular nanosponges inhibit SARS-CoV-2 infectivity. Nano Lett..

[102] Thamphiwatana S., Angsantikul P., Escajadillo T., Zhang Q., Olson J., Luk B.T., Zhang S., Fang R.H., Gao W., Nizet V. (2017). Macrophage-like nanoparticles concurrently absorbing endotoxins and proinflammatory cytokines for sepsis management. Proc. Natl. Acad. Sci. USA.

[103] Higaki M. (2009). Recent development of nanomedicine for the treatment of inflammatory diseases. Inflamm. Regen..

[104] Doshi N., Zahr A.S., Bhaskar S., Lahann J., Mitragotri S. (2009). Red blood cell-mimicking synthetic biomaterial particles. Proc. Natl. Acad. Sci. USA.

[105] Tsai R.K., Rodriguez P.L., Discher D.E. (2010). Self inhibition of phagocytosis: The affinity of “marker of self” CD47 for SIRP alpha dictates potency of inhibition but only at low expression levels. Blood Cells Mol. Dis..

[106] Merkel T.J., Jones S.W., Herlihy K.P., Kersey F.R., Shields A.R., Napier M., Luft J.C., Wu H., Zamboni W.C., Wang A.Z. (2011). Using mechanobiological mimicry of red blood cells to extend circulation times of hydrogel microparticles. Proc. Natl. Acad. Sci. USA.

[107] Xiao F., Fan J., Tong C., Xiao C., Wang Z., Liu B., Daniyal M., Wang W. (2019). An erythrocyte membrane coated mimetic nano-platform for chemo-phototherapy and multimodal imaging. RSC Adv..

[108] Pei L., Petrokivocs I., Way J.L. (1995). Antagonism of the lethal effects of paraoxon by carrier erythrocytes containing phosphotriesterase. Fundam. Appl. Toxicol..

[109] Su J., Sun H., Meng Q., Yin Q., Tang S., Zhang P., Chen Y., Zhang Z., Yu H., Li Y. (2016). Long Circulation red-blood-cell-mimetic nanoparticles with peptide-enhanced tumor penetration for simultaneously inhibiting growth and lung metastasis of breast cancer. Adv. Funct. Mater..

[110] Ansovini R., Compagnucci L. (2020). The hypothetical role of erythrocytes in COVID-19: Immediate clinical therapys. J. Environ. Life Sci..

[111] Wenzhong L., Hualan L. (2020). COVID-19 disease: ORF8 and surface glycoprotein inhibit heme metabolism by binding to porphyrin. chemRxiv.

[112] Ntyonga-Pono M.P. (2020). COVID-19 infection and oxidative stress: An under-explored approach for prevention and treatment?. Pan Afr. Med. J..

[113] Poduri R., Joshi G., Jagadeesh G. (2020). Drugs targeting various stages of the SARS-CoV-2 life cycle: Exploring promising drugs for the treatment of Covid-19. Cell. Signal..

[114] Cavezzi A., Troiani E., Corrao S. (2020). COVID-19: Hemoglobin, iron, and hypoxia beyond inflammation. A narrative review. Clin. Pract..

[115] Bomhof G., Mutsaers P.G.N.J., Leebeek F.W.G., Boekhorst P.A.W., Hofland J., Croles F.N., Jansen A.J.G. (2020). COVID-19-associated immune thrombocytopenia. Br. J. Haematol..

[116] Assinger A. (2014). Platelets and infection—An emerging role of platelets in viral infection. Front. Immunol..

[117] Liu Y., Sun W., Guo Y., Chen L., Zhang L., Zhao S., Long D., Yu L. (2020). Association between platelet parameters and mortality in coronavirus disease 2019: Retrospective cohort study. Platelets.

[118] Xu P., Zhou Q., Xu J. (2020). Mechanism of thrombocytopenia in COVID-19 patients. Ann. Hematol..

[119] Yang M., Ng M.H., Kong Li C., Kong C.L. (2005). Thrombocytopenia in patients with severe acute respiratory syndrome (review). Hematology.

[120] Arnaud C.H. (2016). Platelet disguises could aid drug delivery. Chem. Eng. News.

[121] Korin N., Kanapathipillai M., Ingber D.E. (2013). Shear-responsive platelet mimetics for targeted drug delivery. Isr. J. Chem..

[122] Hu Q., Sun W., Qian C., Wang C., Bomba H.N., Gu Z. (2015). Anticancer platelet-mimicking nanovehicles. Adv. Mater..

[123] Wang H., Wu J., Williams G.R., Fan Q., Niu S., Wu J., Xie X., Zhu L.M. (2019). Platelet-membrane-biomimetic nanoparticles for targeted antitumor drug delivery. J. Nanobiotechnol..

[124] Hu C.M.J., Fang R.H., Wang K.C., Luk B.T., Thamphiwatana S., Dehaini D., Nguyen P., Angsantikul P., Wen C.H., Kroll A.V. (2015). Nanoparticle biointerfacing by platelet membrane cloaking. Nature.

[125] Anselmo A.C., Modery-Pawlowski C.L., Menegatti S., Kumar S., Vogus D.R., Tian L.L., Chen M., Squires T.M., Gupta A.S., Mitragotri S. (2014). Platelet-like nanoparticles: Mimicking shape, flexibility, and surface biology of platelets to target vascular injuries. ACS Nano.

[126] Li R., Liang J., Zhu Y., Qin J. (2018). Drug targeting through platelet membrane-coated nanoparticles for the treatment of rheumatoid arthritis. Nano Res..

[127] Yang G., Chen S., Zhang J. (2019). Bioinspired and biomimetic nanotherapies for the treatment of infectious diseases. Front. Pharmacol..

[128] Almeida J., Edwards D.C., Brand C., Heath T. (1975). Formation of virosomes from influenza subunits and liposomes. Lancet.

[129] Kaneda Y. (2000). Virosomes: Evolution of the liposome as a targeted drug delivery system. Adv. Drug Deliv. Rev..

[130] Daemen T., Demare A., Bungener L., Dejonge J., Huckriede A., Wilschut J. (2005). Virosomes for antigen and DNA delivery. Adv. Drug Deliv. Rev..

[131] Mohammadzadeh Y., Rasouli N., Aref M.H.S., Tabib N.S.S., Abdoli A., Biglari P., Saleh M., Tabatabaeian M., Kheiri M.T., Jamali A. (2016). A novel chimeric influenza virosome containing vesicular stomatitis G protein as a more efficient gene delivery system. Biotechnol. Lett..

[132] Mohammadzadeh Y., Gholami S., Rasouli N., Sarrafzadeh S., Tabib N.S.S., Aref M.H.S., Abdoli A., Biglari P., Fotouhi F., Farahmand B. (2017). Introduction of cationic virosome derived from vesicular stomatitis virus as a novel gene delivery system for sf9 cells. J. Liposome Res..

[133] Liu H., Tu Z., Feng F., Shi H., Chen K., Xu X. (2015). Virosome, a hybrid vehicle for efficient and safe drug delivery and its emerging application in cancer treatment. Acta Pharmaceutica.

[134] Stegmann T., Kamphuis T., Meijerhof T., Goud E., De Haan A., Wilschut J. (2010). Lipopeptide-adjuvanted respiratory syncytial virus virosomes: A safe and immunogenic non-replicating vaccine formulation. Vaccine.

[135] Lederhofer J., Van Lent J., Bhoelan F., Karneva Z., De Haan A., Wilschut J.C., Stegmann T. (2018). Development of a virosomal RSV vaccine containing 3D-PHAD^®^ Adjuvant: Formulation, composition, and long-term stability. Pharm. Res..

[136] De Jonge J., Holtrop M., Wilschut J., Huckriede A. (2005). Reconstituted influenza virus envelopes as an efficient carrier system for cellular delivery of small-interfering RNAs. Gene Ther..

[137] Khoshnejad M., Young P., Toth I., Minchin R. (2007). Modified Influenza virosomes: Recent advances and potential in gene delivery. Curr. Med. Chem..

[138] Saga K., Kaneda Y. (2013). Virosome presents multimodel cancer therapy without viral replication. Biomed Res. Int..

[139] Li H., Tatematsu K., Somiya M., Iijima M., Kuroda S. (2018). Development of a macrophage-targeting and phagocytosis-inducing bio-nanocapsule-based nanocarrier for drug delivery. Acta Biomaterialia.

[140] Matsuura K., Watanabe K., Matsuzaki T., Sakurai K., Kimizuka N. (2010). Self-assembled synthetic viral capsids from a 24-mer viral peptide fragment. Angewandte Chemie Int. Ed..

[141] Hill B.D., Zak A., Khera E., Wen F. (2017). Engineering virus-like particles for antigen and drug delivery. Curr. Protein Pept. Sci..

[142] Smith M.T., Hawes A.K., Bundy B.C. (2013). Reengineering viruses and virus-like particles through chemical functionalization strategies. Curr. Opin. Biotechnol..

[143] Manzenrieder F., Luxenhofer R., Retzlaff M., Jordan R., Finn M.G. (2011). Stabilization of virus-like particles with poly(2-oxazoline) s. Angewandte Chemie.

[144] Steinmetz N.F., Manchester M. (2009). PEGylated viral nanoparticles for biomedicine: The impact of PEG chain length on VNP cell interactions in vitro and ex vivo. Biomacromolecules.

[145] Grgacic E.V.L., Anderson D.A. (2006). Virus-like particles: Passport to immune recognition. Methods.

[146] Kato T., Takami Y., Kumar Deo V., Park E.Y. (2019). Preparation of virus-like particle mimetic nanovesicles displaying the S protein of Middle East respiratory syndrome coronavirus using insect cells. J. Biotechnol..

[147] Ellah N.H.A., Gad S.F., Muhammad K., Batiha G.E., Hetta H.F. (2020). Nanomedicine as a promising approach for diagnosis, treatment and prophylaxis against COVID-19. Nanomedicine.

[148] Wang C., Zheng X., Gai W., Wong G., Wang H., Jin H., Feng N., Zhao Y., Zhang W., Li N. (2017). Novel chimeric virus-like particles vaccine displaying MERS-CoV receptor-binding domain induce specific humoral and cellular immune response in mice. Antiviral Res..

[149] Rohovie M.J., Nagasawa M., Swartz J.R. (2017). Virus-like particles: Next-generation nanoparticles for targeted therapeutic delivery. Bioeng. Transl. Med..

[150] Wu W., Hsiao S.C., Carrico Z.M., Francis M.B. (2009). Genome-free viral capsids as multivalent carriers for taxol delivery. Angewandte Chemie Int. Ed. Engl..

[151] Marcandalli J., Fiala B., Ols S., Perotti M., De van der Schueren W., Snijder J., Hodge E., Benhaim M., Ravichandran R., Carter L. (2019). Induction of potent neutralizing antibody responses by a designed protein nanoparticle vaccine for respiratory syncytial virus. Cell.

[152] Coleman C.M., Liu Y.V., Mu H., Taylor J.K., Massare M., Flyer D.C., Smith G.E., Frieman M.B. (2014). Purified coronavirus spike protein nanoparticles induce coronavirus neutralizing antibodies in mice. Vaccine.

